# Pre-pregnancy body mass index classification and gestational weight gain on neonatal outcomes in adolescent mothers: A follow-up study

**DOI:** 10.1371/journal.pone.0200361

**Published:** 2018-07-12

**Authors:** Reyna Sámano, Gabriela Chico-Barba, Hugo Martínez-Rojano, Estela Godínez, Ana Lilia Rodríguez-Ventura, Gabriela Ávila-Koury, Karen Aguilar-Sánchez

**Affiliations:** 1 Departamento de Nutrición y Bioprogramación, Instituto Nacional De Perinatología “Isidro Espinosa de Los Reyes”, Ciudad de México, México; 2 Escuela de Enfermería, Facultad de Ciencias de la Salud, Universidad Panamericana, Ciudad de México, México; 3 Sección de Posgrado e Investigación, Escuela Superior de Medicina, Instituto Politécnico Nacional, Ciudad de México, México; 4 Coordinación de Medicina Laboral, Instituto de Diagnóstico y Referencia Epidemiológico “Manuel Martínez Báez”, Ciudad de México, México; 5 Licenciatura en Nutrición y Ciencias de los Alimentos, Universidad Iberoamericana, Ciudad de México, México; 6 Instituto Mexicano del Seguro Social, Tlaxcala, México; Centre Hospitalier Universitaire Vaudois, FRANCE

## Abstract

Introduction: Institute of Medicine gestational weight gain recommendations are based on body mass index (BMI) status using adult cut-off points for women of all ages, even though adolescents have specific criteria, like WHO and CDC, so adolescents can receive inadequate weight gain recommendations. Objectives: To estimate the proportion of classification disparity between the three criteria (WHO, CDC and IOM) of pre-pregnancy BMI status; and to analyze neonatal outcomes according to weight gain recommendation based on pre-pregnancy BMI using the three criteria. Methods: Follow-up study in pregnant adolescents 12–19 years. Sociodemographic, anthropometric and pregnancy data were obtained. Percentage of pre-pregnancy BMI classification disparity was calculated between three criteria. Gestational weight gain was categorized in adequate, low and high according to IOM. Regression models were used to analyze negative neonatal outcomes. Results: 601 pregnant adolescents were included, mean age was 16±1.4 years. For pre-pregnancy BMI classification, 28.5% had classification disparity using IOM vs WHO, and 14% when comparing IOM vs CDC. Greater classification disparity was observed as BMI increased. When using WHO categories, a high weight gain was associated with increased risk of having a low birth weight baby (OR: 1.91, CI95%: 1.03–3.53). For CDC criteria, a low weight gain was associated with increased risk of having a preterm baby (OR: 2.65; CI95%: 1.16–6.08) and a high weight gain was associated with low birth weight (OR: 2.10; CI95%: 1.10–4.01). For IOM criteria, a weight gain either low or high were associated with increased risk of low birth weight and preterm birth. Conclusion: There is pre-pregnancy BMI classification disparity using criteria for adolescents compared to adult criteria. Nevertheless, with WHO and CDC only a high gestational weight gain was a risk for negative neonatal outcome. It is important to have a BMI classification system for adolescents that better predicts neonatal outcomes.

## Introduction

Adolescent pregnancy is a Public Health problem in Mexico, its rate is one the highest in Latin America (64 out of 1000 pregnancies [1]; it can lead to maternal and neonatal negative outcomes like higher risk of preeclampsia, low birth weight and preterm birth [2]. Gestational weight gain (GWG) is involved in some of these negative outcomes. In the United States, according to the Institute of Medicine (IOM) recommendations for weight gain during pregnancy [3], 17% of pregnant adolescents had an adequate weight gain and 57.2% had an excessive weight gain; even more, a low weight gain was related to higher risk of small for gestational age (SGA) regardless of pre-pregnancy body mass index (BMI) [4]. It was reported that accomplishing GWG recommendation by the IOM, decreased the frequency of macrosomia, gestational hypertension, preeclampsia and cesarean section, although there are more factors related to these outcomes [5]. IOM weight gain recommendations are based on BMI status using adult cut-off points for women of all ages, even though adolescents have specific categories for them using percentiles derived from either World Health Organization (WHO) [6] or Center for Disease Control (CDC) growth charts BMI for age and sex [7]. Pre-pregnancy BMI classification determines the prescription of GWG, which has direct clinical impact on outcomes. In this way, when IOM criteria is used for pre-pregnancy BMI status for adolescents, their BMI category is sometimes underestimated, so these girls will receive a wider range of weight gain recommendation, leading to an excessive weight gain during pregnancy, adverse maternal and neonatal outcomes and postpartum weight retention [8]. We, therefore, hypothesized that classification disparity would be present using IOM criteria for pre-pregnancy BMI classification and that a higher risk of neonatal outcomes according to gestational weight gain would be present using IOM criteria.

The aims of this study in a sample of pregnant adolescents were 1) to estimate the proportion of classification disparity between the three criteria (WHO, CDC and IOM) in the assessment of pre-pregnancy BMI status; and 2) to analyze neonatal outcomes according to weight gain recommendation based on her pre-pregnancy BMI status using the three different criteria.

## Methods

### Study design

This was a prospective cohort study including pregnant adolescents from 12 to 19 years old that were attended for prenatal care at the Instituto Nacional de Perinatología (National Perinatology Institute, INPer) in Mexico City, for the period between 2013 to 2016. Sampling was non-probabilistic, based on consecutive cases that complied the following inclusion criteria: weight and height measured before and at the end of the study; gestational age according to last menstrual period; not to have chronic or infectious diseases; first and singleton pregnancy; from Mexico City and states nearby; and written informed form consent from adolescents, as well as from their parents or guardians. During this period, a total of 800 adolescents met the inclusion criteria, but only 601 (75%) accepted to participate. Sample size was calculated according to 57% of reported adverse outcomes[9], requiring a total of 377 participants. Nevertheless, a total of 601 participants were included in the study. This number was intended to over-represent all of pregnant adolescents that had prenatal medical care at INPer and to avoid statistical error. In order to control possible selection bias, sociodemographic and clinical characteristics from the participants were compared to those who did not meet inclusion criteria; no statistical significant differences were observed between groups.

### Sociodemographic data

Age, education, occupation and socioeconomic status were obtained through a questionnaire at the baseline assessment.

### Anthropometric assessment

Standardized personnel using the Lohman technique performed the anthropometric measurements. Lohmann's technique consists of the standardization of the procedures for the realization of the different anthropometric measurements, which was developed by Lohman in 1988 [10]. Pregestational weight was self-reported, then, it was compared to the weight stated in the medical record. In adolescents, self-reported weight and height are reliable and highly correlate with real measurements [11]. Final weight was obtained one week before birth, between 7 and 9 am, fasting, using a digital scale (Tanita BVB-600, 0.1 kg of precision). Height was assessed using a stadiometer (SECA 274, 0.1 cm of precision).

### BMI classification

Pre-pregnancy BMI was calculated by dividing pre-pregnancy weight in kilograms by the height in squared meters (weight/height^2^) and then categorized according to three different criteria: 1) percentiles derived from the World Health Organization growth charts for BMI for sex and age (hereafter referred to as WHO) [6]; 2) percentiles derived from the Center for Disease Control growth charts for BMI for age and sex (from now on referred to as CDC) [7]; and 3) guidelines for weight gain during pregnancy from the Institute of Medicine according to pre-pregnancy BMI (hereafter referred IOM) [3], as seen in Table 1. Classification disparity was defined as the erroneous classification of pre-pregnancy BMI category when comparing two different criteria: (WHO *vs* IOM and CDC *vs* IOM).

**Table 1:** Body mass index categories according to three different criteria.

	WHO^a^	CDC^b^	IOM^c^
**Underweight**	<3rd	<5th	<18.5
**Normal Weight**	3rd-<85th	5th-<85th	18.5–24.9
**Overweight**	85th-<97th	85th-<95th	25.0–29.9
**Obese**	≥97th	≥95th	≥30.0

^a^ BMI Percentiles for sex and age, WHO [6]

^b^ BMI Percentiles for sex and age, CDC [7]

^c^ BMI for adults (kg/m^2^), IOM [3]

### Gestational weight gain

GWG was referred as the difference between maternal weight one week before delivery and pre-pregnancy weight. Recommended GWG was calculated based on Institute of Medicine recommendations according to pre-pregnancy BMI: underweight a gain of 12.5–18 kg; normal weight a gain of 11.5–16 kg; overweight a gain of 7–11.5 kg; and obese a gain of 5–9 kg[12]. After this, GWG was divided into three categories: low, if the weight was below the recommendation; adequate, if the weight gain was within the recommendation; and high, if the weight gain was above the recommendation. GWG categories were determined using the three different criteria to classify pre-pregnancy BMI.

### Neonatal characteristics

Three neonatal outcomes were assessed using medical records: 1) low birth weight (LBW), <2500g; 2) preterm birth, ≤36 gestational weeks; and 3) small for gestational age (SGA) according to weight for gestational age of Mexican children [13].

### Ethical considerations

The Institutional Review Board and Ethics Committee from Instituto Nacional de Perinatología approved the study (No. 212250–49481). Data gathering was confidential, taking ethical questions such as autonomy and security into account. The guidelines of The Helsinki Declaration were followed. All adolescents received medical attention at INPer.

### Statistical analysis

We performed a descriptive analysis on the characteristics of the study population. Frequencies and percentages were calculated for categorical variables, and mean and standard deviation for continuous variables. The percentage of pre-pregnancy BMI classification disparity was calculated by cross-tabulating the proportion of participants in each WHO and CDC category with the proportion of each IOM category (WHO vs IOM and CDC vs IOM). The classification disparity was stratified by age group (12–15 years and 16–19 years) because some studies report that there is a greater disparity in pre-pregnancy BMI classification in younger adolescents and that adolescents older than 16 years have pregnancy characteristics similar to adults [14,15]. GWG category was compared to pre-pregnancy BMI by cross-tabulation. Logistic regression was performed to obtain odds ratio with 95% confidence intervals for the associations between GWG and neonatal outcomes. Regression models were adjusted for variables that showed statistical significance in the bivariate analysis: pre-pregnancy BMI, maternal age and socioeconomic status. All statistical analyses were carried out using IBM SPSS Statistics for Windows, Version 20.0. (Armonk, NY: IBM Corp). Statistical significance was considered at p < 0.05.

## Results

### General characteristics

A total of 601 adolescents and their children were included in the analysis; the characteristics of the sample are presented in Table 2. Mean age was 16±1.4 years, 39.1% (n = 235) were 12 to 15 years and 60.9% (n = 366) were 16 and older. The most common occupation was home duties (76.7%, n = 461). Mean pre-pregnancy BMI was 21.5±3.3 kg/m^2^. Overall, the proportion of preterm, low birth weight and small for gestational age were 10.3%, 15.8% and 17.3%, respectively. The frequency of preeclampsia was not different between preterm and term (p = 0.824), low and normal birth weight (p = 0.239) and small and adequate for gestational age (p = 0.344). Also, socioeconomic status did not differ between the neonatal outcomes.

**Table 2:** Characteristics of the studied population (n = 601).

Variable	n (%)
**Education**	
None	10 (1.7)
Elementary school	145 (24.1)
Junior high	369 (61.4)
High school	74 (12.3)
College	3 (0.5)
**Occupation**	
Home duties	461 (76.7)
Student	109 (18.2)
Employed	15 (2.5)
Self-employed	16 (2.6)
Socioeconomic status	
Low	260 (43.3)
Medium	237 (39.4)
High	104 (17.3)
**Prenatal Care Initiation**	
First trimester	205 (34.1)
Second trimester	332 (55.2)
Third trimester	64 (10.6)
C-section	279 (46.4)
Weight gain (kg)^a^	12.3 (±5.9)
**Neonate**	
Gestagional Age (wk)^a^	38 (±1.7)
Birth Weight (kg)^a^	2904 (±466)
Birth Length (cm)^a^	48.6 (±2.6)
Low birth weight (<2500g)	95 (15.8)
Preterm (<37 wk)	62 (10.3)
Small for gestational age	104 (17.3)

^a^ Mean (± Standard Deviation)

### Pre-pregnancy BMI classification

**Fig 1:**
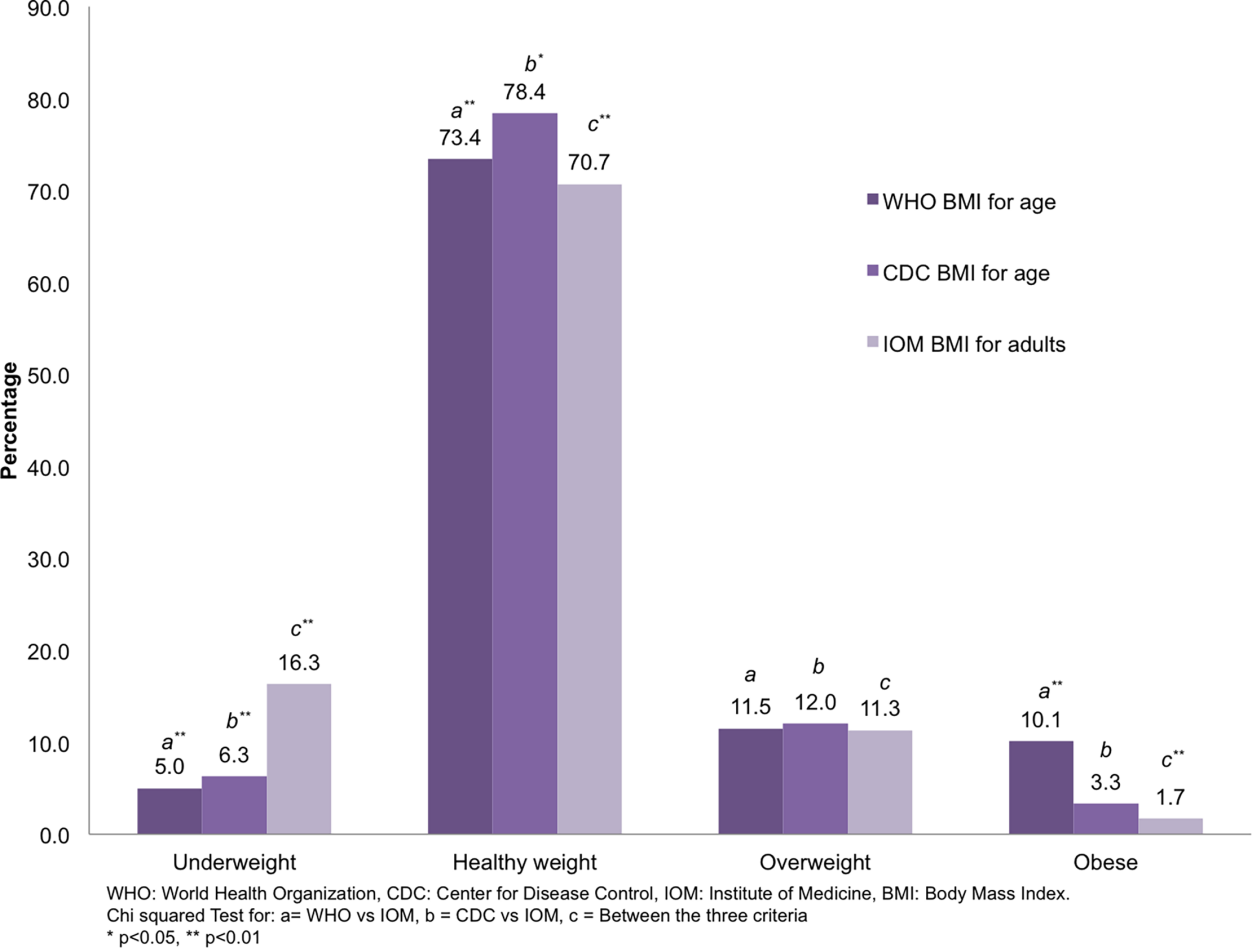
Pre-pregnancy BMI according to three different criteria.

According to WHO criteria for adolescents, the proportion of overweight and obese girls was 11.5% (n = 69) and 10.1% (n = 61), respectively; with CDC criteria the proportions were similar for overweight (12%, n = 72) and lower for obesity (3.3%, n = 20); using IOM criteria for adults, overweight was 11.3% (n = 68) an obesity 1.7% (n = 10). The frequency for underweight was 5.0% (n = 30), 6.3% (n = 38) and 16.3% (n = 98), for WHO, CDC and IOM criteria, respectively. (Fig 1).

When comparing WHO categories with the IOM categories, 28.5% (n = 171) of the sample had classification disparity. As the BMI category increased the higher percentage of classification disparity occurred. Similar results were obtained when using CDC categories compared to IOM categories; a total of 14% (n = 84) had classification disparity and as BMI category increased the higher percentage of classification disparity occurred. However, using either WHO or CDC, all underweight adolescents were correctly classified.

Higher classification disparity was observed in younger adolescents (12–15 years old) when compared to older (16–19 years old). The proportion of classification disparity was around 24% in both age groups using WHO criteria compared to IOM. Nevertheless, when using CDC criteria, more number of young adolescents had classification disparity compared to older (21.7% vs 9%, respectively) (Table 3). These results are presented in a table format used by other authors previously [8,16].

**Table 3:** Pre-pregnancy BMI classification disparity between adolescent vs adult categories.

Adolescent Pre-pregnancy BMI		Adult	All	12–15 y	16–19 y
		IOM categories	n (%)	n (%)	n (%)
**WHO categories**^**a**^	**Underweight**	Low (correctly classified)	30 (100.0)	10 (100.0)	20 (100.0)
	**(n = 30)**	Normal (disparity)	0	0	0
		Overweight (disparity)	0	0	0
		Obese (disparity)	0	0	0
	**Healthy weight**	Low (disparity)	68 (15.4)	32 (18.6)	36 (13.4)
	**(n = 441)**	Normal (correctly classified)	373 (84.6)	140 (81.4)	233 (86.6)
		Overweight (disparity)	0	0	0
	** **	Obese (disparity)	0	0	0
	**Overweight**	Low (disparity)	0	0	0
	**(n = 69)**	Normal (disparity)	52 (75.4)	27 (100.00)	25 (59.5)
		Overweight (correctly classified)	17 (24.6)	0	17 (40.5)
	** **	Obese (disparity)	0	0	0
	**Obese**	Low (disparity)	0	0	0
	**(n = 61)**	Normal (disparity)	0	0	0
		Overweight (disparity)	51 (83.6)	24 (92.3)	27 (77.1)
	** **	Obese (correctly classified)	10 (16.4)	2(7.7)	8 (22.9)
**CDC categories**^**b**^	**Underweight**	Low (correctly classified)	38 (100.0)	10 (100.0)	28 (100.0)
	**(n = 38)**	Normal (disparity)	0	0	0
		Overweight (disparity)	0	0	0
	** **	Obese (disparity)	0	0	0
	**Healthy weight**	Low (disparity)	60 (12.7)	32 (16.9)	28 (9.9)
	**(n = 471)**	Normal (correctly classified)	411 (87.3)	157 (83.1)	254 (90.1)
		Overweight (disparity)	0	0	0
	** **	Obese (disparity)	0	0	0
	**Overweight**	Low (disparity)	0	0	0
	**(n = 72)**	Normal (disparity)	14 (19.4)	10 (40.0)	4 (8.5)
		Overweight (correctly classified)	58 (80.6)	15 (60.0)	43 (91.5)
	** **	Obese (disparity)	0	0	0
	**Obese**	Low (disparity)	0	0	0
	**(n = 20)**	Normal (disparity)	0	0	0
		Overweight (disparity)	10 (50.0)	9 (81.8)	1 (11.1)
	** **	Obese (correctly classified)	10 (50.0)	2 (18.2)	8 (88.9)

^a^ Percentages presented by column and WHO category. WHO vs IOM, Kappa = 0.379, p<0.01

^b^ Percentages presented by column and CDC category. CDC vs IOM, Kappa = 0.668, p<0.01

### Gestational weight gain

**Fig 2:**
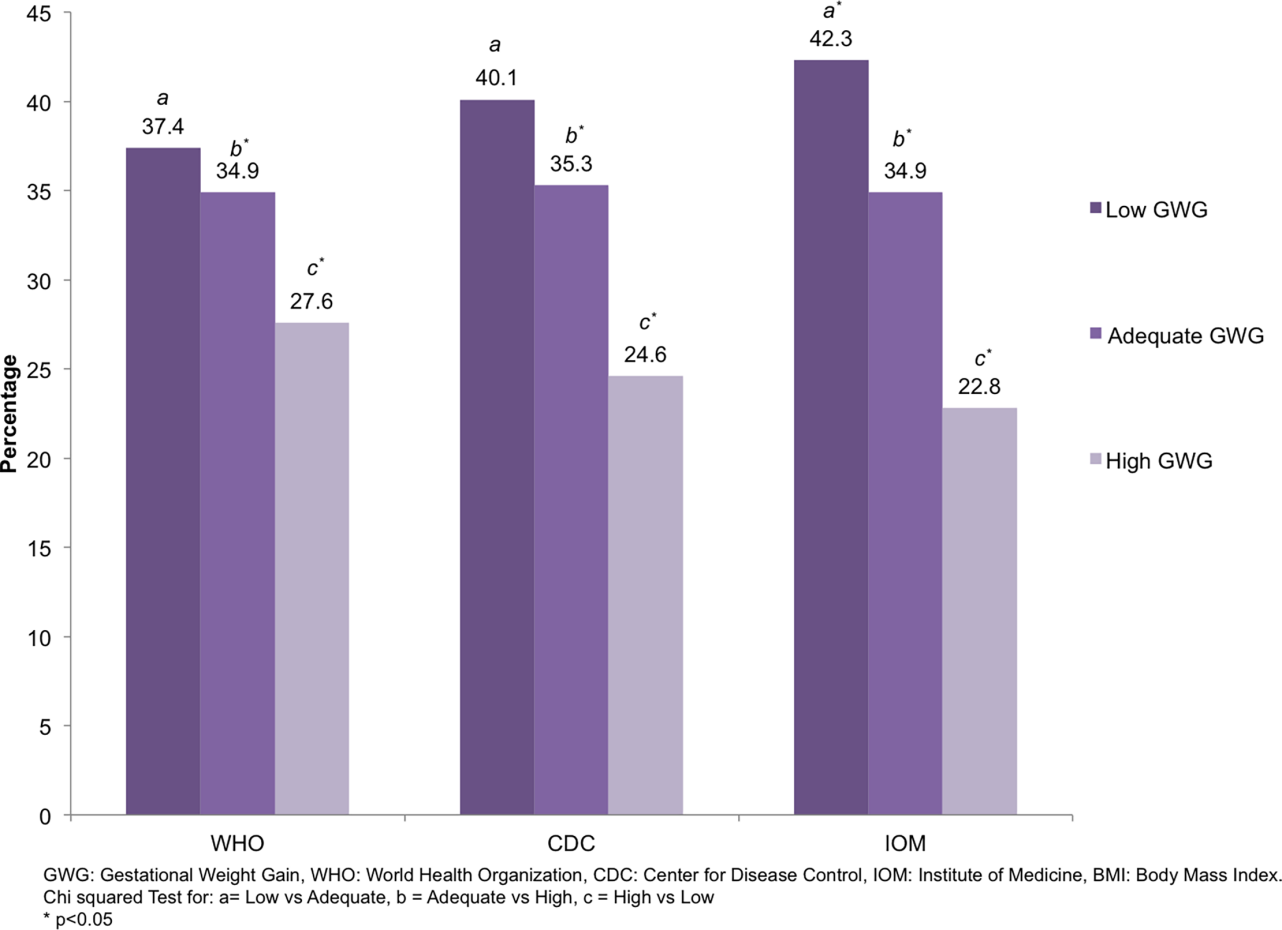
Gestational weight gain according to IOM recommendation, using three different criteria of pre-pregnancy BMI classification.

Overall, mean GWG was 12.3±5.9 kg. In accordance with IOM recommendations about 42% and 23% of the adolescents had low and high GWG, respectively. These proportions were different when using WHO and CDC criteria (Fig 2). GWG by pre pregnancy BMI status is summarized in Table 4; most obese adolescents had weight gain above the recommendation, regardless of the criteria used for pre pregnancy BMI.

**Table 4:** Gestational weight gain according to IOM recommendation, by pre-pregnancy BMI classification.

Pre-pregnancy BMI classification	Low GWG	Adequate GWG	High GWG	p-value
	n (row %)	n (row %)	n (row %)	
**WHO**				<0.01
**Underweight**	11 (36.7)	12 (40.0)	7 (23.3)	
**Healthy weight**	184 (41.7)	163 (37.0)	94 (21.3)	
**Overweight**	12 (17.4)	24 (34.8)	33 (47.8)	
**Obese**	18 (29.5)	11 (18.0)	32 (52.5)	
**CDC**				<0.01
**Underweight**	19 (50.0)	12 (31.6)	7 (18.4)	
**Healthy weight**	198 (42.0)	171 (36.3)	102 (21.7)	
**Overweight**	18 (25.0)	27 (37.5)	27 (37.5)	
**Obese**	6 (30.0)	2 (10.0)	12 (60.0)	
**IOM**				0.005
**Underweight**	45 (45.9)	33 (33.7)	20 (20.4)	
**Healthy weight**	187 (44.0)	152 (35.8)	86 (20.2)	
**Overweight**	18 (26.5)	24 (35.3)	26 (38.2)	
**Obese**	4 (40.0)	1 (10.0)	5 (50.0)	

Percentages presented per rows.

### Neonatal outcomes

**Fig 3:**
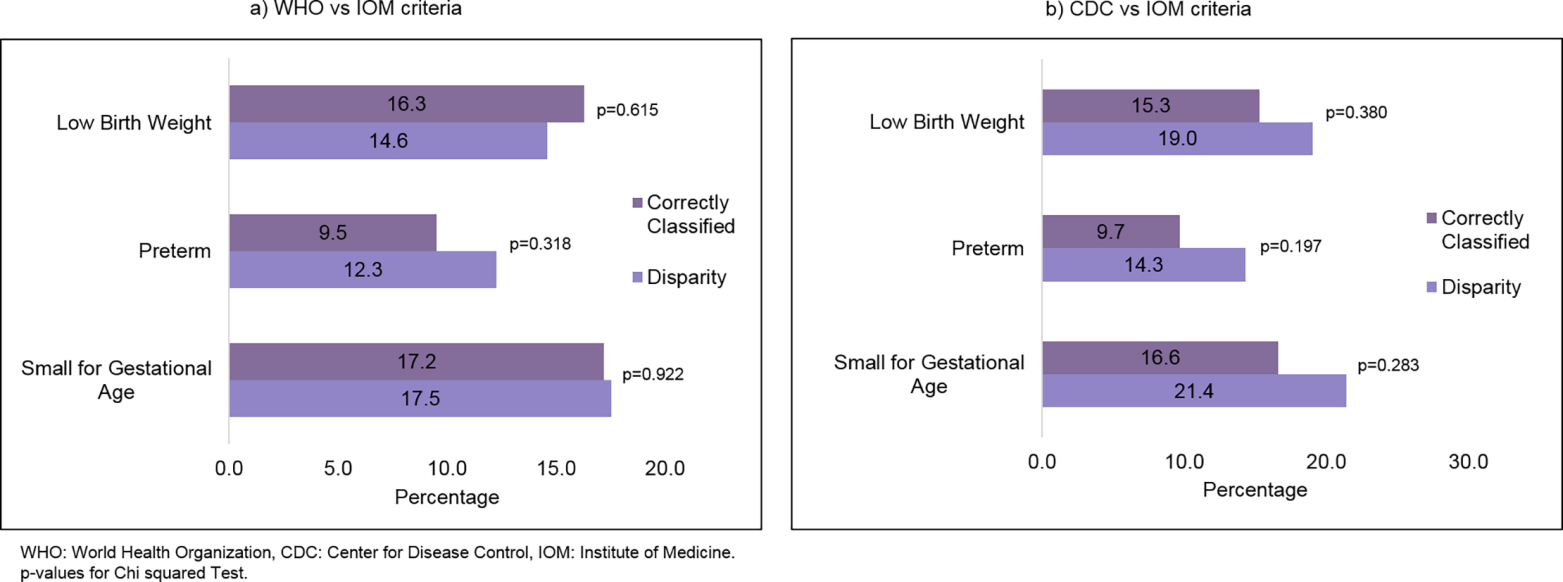
Distribution (%) of neonatal outcomes in adolescents with a pre-pregnancy BMI correctly classified compared to those with disparity classification, according to different criteria of pre-pregnancy BMI classification.

Neonatal outcomes in adolescents with classification disparity are shown in Fig 3. There was no differences in percentages of LBW, preterm and SGA when compared to correctly classified adolescents.

The multivariate logistic regressions for LBW, preterm and SGA are presented in Table 5. When using WHO categories, a high GWG was associated with increased risk of having a baby of LBW (Odds Ratio (OR): 1.91, Confidence Interval (CI95%): 1.03–3.53). For CDC criteria, a low GWG was associated with increased risk of having a preterm baby (OR: 2.65; CI95%: 1.16–6.08) and a high GWG was associated with LBW (OR: 2.10; CI95%: 1.10–4.01). For IOM criteria, a GWG either low or high were associated with increased risk of LBW and preterm.

**Table 5:** Associations between neonatal outcomes and gestational weight gain using pre-pregnancy BMI.

Pre-pregnancy BMI classification	GWG	LBW (n = 95)	Preterm (n = 62)	SGA (n = 153)
		aOR (IC95%)	aOR (IC95%)	aOR (IC95%)
**WHO criteria**	**Adequate (n = 210)**	Ref.	Ref.	Ref.
	**Low (n = 225)**	1.47 (0.77–2.78)	1.76 (0.83–3.69)	1.04 (0.57–1.90)
	**High (n = 166)**	1.91 (1.03–3.53)	1.56 (0.74–3.27)	0.60 (0.34–1.05)
**CDC criteria**	**Adequate (n = 212)**	Ref.	Ref.	Ref.
	**Low (n = 241)**	1.73 (0.89–3.38)	2.65 (1.16–6.08)	0.83 (0.45–1.53)
	**High (n = 148)**	2.10 (1.10–4.01)	2.15 (0.93–4.96)	0.57 (0.32–1.02)
**IOM criteria**	**Adequate (n = 210)**	Ref.	Ref.	Ref.
	**Low (n = 254)**	2.10 (1.02–4.32)	2.61 (1.09–6.27)	0.71 (0.37–1.35)
	**High (n = 137)**	2.66 (1.32–5.33)	2.61 (1.10–6.19)	0.52 (0.28–0.96)

GWG: Gestational weight gain; LBW: Low birth weight; SGA: Small for gestational age; aOR: Adjusted Odds Ratio for pre-pregnancy BMI, maternal age and socioeconomic status; CI: Confidence interval.

## Discussion

In this study, 601 pregnant adolescents who attended the INPer were evaluated. Although the data is not from a representative sample of the country, most of these adolescents who attend the institute come from a low socioeconomic level and have no health care insurance. In Mexico, it has been reported that within this socioeconomic level the prevalence of pregnancy in adolescence is the highest [17,18]; for this reason, the results of this study could be extrapolated to more pregnant adolescents in the country and in Latin America.

Inadequate gestational weight gain and negative perinatal outcomes may occur more frequently in groups of pregnant adolescents with lower income, as reported in the Brazilian [19] and South African [20] populations. In Mexican population the information related to adolescent pregnancy and its outcomes is scarce, there is only descriptive data regarding birth weight [21], while Mexico has the second place of pregnancy in adolescents in Latin America. It is important to note that the phenotype of Mexican and Latino adolescent girls is different from American adult women, who are the reference of the GWG recommendations according to the IOM, and therefore Mexican adolescents belong to a different ethnic group [22]. When we used IOM recommendations for GWG in this group, the GWG is greater than expected; similar results have also been reported by Fernández *et a*.*l* [8], who described the discordance between CDC and IOM criteria to classify pre-pregnancy BMI in pregnant adolescents; and the study performed by Barrios *et al*. [23], in a group of Brazilian pregnant adolescents, Barrios *et al*. assessed pre-pregnancy BMI using WHO, IOM and the Ministry of Health of Brazil criteria and concluded that criteria from the Ministry of Health of Brazil should be used to assess pregnant adolescents.

The findings in this study support the discussion on the choice of the best criterion to classify pre-pregnancy BMI and the recommendation of GWG in the prenatal care of pregnant adolescents. Taiwo *et al*. [24] studied a group of pregnant adolescents and adults from Nigeria, the authors concluded that, despite of having a GWG higher than adults, the recommendation of GWG in adolescents should be reconsidered because they had more risk of having a newborn with LBW.

The pre-pregnancy BMI of over a quarter of the participants had classification disparity when comparing WHO vs IOM criteria. When using CDC vs IOM criteria, 14% of the sample had classification disparity; similar results were observed by Amaral *et al*. [16]. IOM guidelines for GWG do not have a specific weight gain recommendation for younger women; in this way, there is a trend to overestimate the percentage of underweight in pregnant adolescents, especially in those aged 15 and younger. In addition, the higher the BMI the higher classification disparity, as previous studies have reported [8,16]. However, the great proportion of classification disparity in younger adolescents (≤15 years) is not surprising, since older adolescents are more likely to have sexual maturation than younger adolescents, so they are closer to the body mass of an adult woman [8]. All adolescents with a pre-pregnancy BMI category of underweight were correctly classified by the IOM criteria. After almost 10 years of these results[8], there is no precise guidance for health care providers on the optimal gestational weight gain in adolescents, especially for those providers in first-contact health services. The consequence of a pre-pregnancy BMI classification disparity is an inadequate prescription of GWG, with consequent negative outcomes, like postpartum weight retention. This is of concern, since the prevalence of overweight and obesity in Mexican female adolescents is around to 38% [25], which can also affect the weight of the newborn and other perinatal outcomes.

In this study, about 25% of the participants had an adequate GWG, this was similar than the 28% reported in Thailand adolescents [26]; this low percentage can be related to a greater frequency of negative perinatal outcomes, like preterm birth and LBW. In Mexican population, it has been reported that regardless of GWG, adolescents with a healthy pregnancy have higher risk of having a baby with LBW [15].

Although Fernández *et al*. since 2011 [8] said that IOM recommendations for GWG should be used for women of all ages, it is contrary to the findings of this study. It has been reported that high or inadequate GWG in adolescent women can be related to overweight, even 18 years after the pregnancy [27], as it happens in adult women. The postpartum retention can lead to non-communicable diseases, as a result of a high pre-pregnancy BMI and high GWG [28].

Regarding neonatal outcomes in this study, the proportion of LBW, preterm and SGA were 15.8, 10.3 y 17.3%, respectively; this numbers are higher than others reported in the Latin American region. According to the Mexican National Health and Nutrition Survey 2012, the prevalence of LBW was 8.37% [29]; in Latin America the prevalence of preterm birth is 8.1% [30]; and the percentage of SGA in Mexico is 6% [31]. The differences between the percentages from this study and the mentioned for Mexico and Latin America may be due to the fact that the percentages in Mexico and Latin America include women of all ages, so, in adolescents, the frequency of these neonatal outcomes is higher. In addition, in this study it was observed that preterm and LBW outcomes were associated with high or low GWG; the adolescents that had greater GWG than recommended, were less likely to have children who were SGA. Similar results were shown by Harper *et al*. [32]; according to the findings from this study, pre-pregnancy BMI classification using CDC criteria has very similar outcomes as the IOM classification [3].

A correct pre-pregnancy BMI classification and its adequate GWG recommendation in pregnant adolescents, contribute to timely implementation of interventions to improve mother and neonate health, especially in those who are overweight or obese. Other studies about pre-pregnancy BMI and its GWG, have shown that an adequate GWG decreases neonatal mortality rates and preterm births, and is also a protective factor for having a macrosomic newborn and for postpartum weight retention; the last two are considered risk factors for cardiovascular diseases [8,33].

A limitation of this study is that most of the pregnant adolescents who participated in come from a low socioeconomic status, so it would be important to include participants from the other strata and compare the results. Another limitation is that the majority of participants had a normal pre-pregnancy BMI category and few of them were overweight/obese (20%, 13% and 12% for WHO, CDC and IOM criteria, respectively), even though in Mexico the prevalence of overweight/obesity in adolescents is 35% [25]. During the recruitment process, there was an especial emphasis to invite to participate adolescents with higher BMI. Nevertheless, there were no sufficient adolescents with overweight/obesity as we expected. This could be explained by biological and behavioural factors. For the biological factors, it is has been reported that the greater the BMI the less fertility in adult and adolescent women [34]. For the behavioural factors, in adults, obese women are less likely than normal-weight women to have a sexual partner during the last 12 months [35], and in adolescents, it is reported that adolescents with overweight/obesity delay their sexual initiation age [36].

## Conclusions

Regarding to pre-pregnancy BMI classification, when compared to IOM, there is more classification disparity with WHO criteria than with CDC criteria. For both pre-pregnancy BMI classification criteria for adolescents, greater classification disparity was observed in adolescents with the highest BMI and in the group of 12 to 15 years. Using both WHO and CDC criteria and regardless of pre-pregnancy BMI, a high gestational weight gain is associated with a greater risk of low birth weight. When using IOM criteria, inadequate gestational weight gain, whether high or low, is associated with negative neonatal outcomes, except for small for gestational age.

As a recommendation, it is necessary to continue with the line of research in order to have an appropriate classification criterion for this age group that better adjust to optimal neonatal outcomes, using the recommended gestational weight gain already established. This is important especially for adolescents under 15 years of age, because a high gestational weight gain recommendation may increase the risk of negative outcomes in the mother and the newborn and can lead to postpartum weight retention.
